# The Delivery of Extracellular Vesicles Loaded in Biomaterial Scaffolds for Bone Regeneration

**DOI:** 10.3389/fbioe.2020.01015

**Published:** 2020-08-19

**Authors:** Hui-Chun Yan, Ting-Ting Yu, Jing Li, Yi-Qiang Qiao, Lin-Chuan Wang, Ting Zhang, Qian Li, Yan-Heng Zhou, Da-Wei Liu

**Affiliations:** ^1^Department of Orthodontics, Peking University School and Hospital of Stomatology, Beijing, China; ^2^National Clinical Research Center for Oral Diseases & National Engineering Laboratory for Digital and Material Technology of Stomatology, Beijing, China; ^3^Beijing Key Laboratory of Digital Stomatology, Beijing, China; ^4^Department of Stomatology, The First Affiliated Hospital of Zhengzhou University, Zhengzhou, China; ^5^Eastman Institute for Oral Health, University of Rochester, Rochester, NY, United States

**Keywords:** extracellular vesicle, tissue scaffold, bone regeneration, tissue engineering, regenerative medicine

## Abstract

Extracellular vesicles (EVs) are heterogeneous nanoparticles actively released by cells that comprise highly conserved and efficient systems of intercellular communication. In recent years, numerous studies have proven that EVs play an important role in the field of bone tissue engineering (BTE) due to several advantages, such as good biosafety, stability and efficient delivery. However, the application of EVs therapies in bone regeneration has not been widely used. One of the major challenges for the application of EVs is the lack of sufficient scaffolds to load and control the release of EVs. Thus, in this review, we describe the most advanced current strategies for delivering EVs with various biomaterials for the use in bone regeneration, the role of EVs in bone regeneration, the distribution of EVs mediated by biomaterials and common methods of promoting EVs delivery efficacy with a focus on biomaterial properties.

## Introduction

**FIGURE 1:**
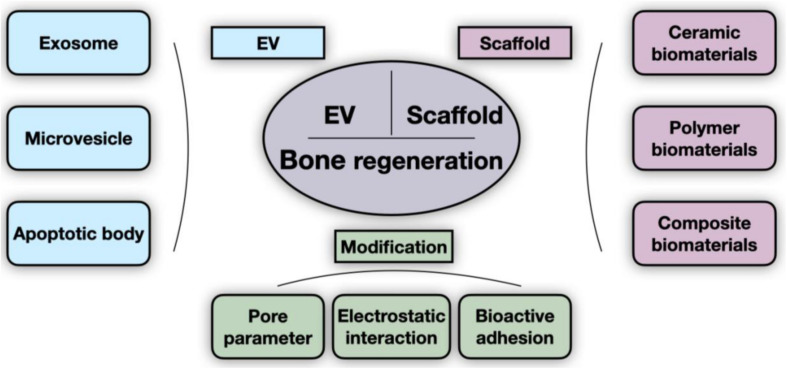
General scheme of scaffold-delivered EVs therapies. The innovative bone regeneration strategy is based on the osteogenic effect of EVs and scaffolds. Extracellular vesicles (EVs) are grouped into three categories (exosome, microvesicle, and apoptotic body), as indicated. The scaffolds described in existing studies consist of ceramic, polymer, and composite biomaterials. Pore parameters (such as porosity and pore size), electrostatic interaction, and bioactive adhesion are currently the main parameters that are modified to improve delivery efficiency.

Extracellular vesicles (EVs) are membrane-encapsulated heterogeneous particles actively released by cells that comprise highly conserved and efficient systems of intercellular communication. EVs have progressed from the original conception as “platelet dust” to a powerful tool with broad biological functions. EVs have become a widespread topic of interest in tissue homeostasis, immune modulation, and metastasis of tumors (Wiklander et al., 2019). The distinct origins of EVs biogenesis account for differences in the markers and contents in EVs, and the compositions of EVs are a reflection of the physiological and pathological state of the host cells and change depending on the stress state and microenvironment (Andaloussi et al., 2013). It has been reported that mesenchymal stem cell (MSC)-derived EVs showed compatible regenerative potential when compared with MSCs (Jong et al., 2014; Zhang et al., 2016; Crivelli et al., 2017; Presen et al., 2019; Yang et al., 2020). Thus, as a potential alternative for tissue engineering, EVs are even more attractive than stem cell transplantation due to several advantages, such as good biosafety, stability, and efficient delivery (Eleuteri and Fierabracci, 2019). However, one of the major challenges of the use of EVs therapies in bone tissue engineering (BTE) is that free EVs do not allow durable retention at defect sites, since it is difficult for EVs to achieve sustained aggregation and controlled release without proper scaffold support; in addition, they are not able to escape from clearance by the immune system, which led to the consideration of scaffolds as carriers for loading EVs (Pinheiro et al., 2018; Zhang K. et al., 2018). To date, there have been an increasing number of studies of biomaterial-mediated EV therapies, in which diverse scaffolds with modifications of their properties have shown great potential for loading and controlling the release of EVs. Although several recent reviews have focused on EV application in tissue regeneration as well as the involved mechanisms of EVs (Chen et al., 2017; Silva et al., 2017; Taverna et al., 2017; Keshtkar et al., 2018; Li Q. et al., 2018; Chu et al., 2019), our review specifically focuses on the controlled release of EVs loaded in scaffolds and provides a discussion of the existing and potential modifications of EVs delivery systems for BTE (Figure 1).

## Current State of Tissue Engineering-Based Bone Regeneration

### Trends in Bone Tissue Engineering

Repair of bone defects has historically been a challenge for both patients and doctors. Over the past few decades, therapeutic strategies for bone defect repair have been evolutionarily updated. The traditional clinical treatments of bone deficiency mainly utilize bone grafting. However, autograft treatment is limited by bone volume, poor availability, donor site damage and other complications (Deng et al., 2018; Zhang Y. et al., 2018), while allografts frequently lead to an increase in the risks of disease transmission, vascularization problems, and immunological rejection (Einhorn and Gerstenfeld, 2015; Vanderstappen et al., 2015). To overcome these problems, BTE, which involves scaffolds, bioactive substances and cells/tissues with osteogenic potential (Wong et al., 2010), has been adapted as a more feasible and sustainable treatment approach for bone regeneration.

Cells employed in BTE play crucial roles in bone regeneration but facing challenges in their use as well. Characterized by their capacities for self-renewal and multipotent differentiation, stem cells are recognized as a well-accepted option in the field of cell-based therapy (Vizoso et al., 2017). Among all types of stem cells, MSCs have attracted considerable attention for their capacity to regulate cell and tissue homeostasis without the risk of cellular immunological rejection or tumor formation, and they have been broadly used in BTE (Le Blanc et al., 2008; Liu et al., 2011; Akiyama et al., 2012; Gomzikova and Rizvanov, 2017; Shi et al., 2017; Chen et al., 2018). However, the inherent risks of the functional engraftment of tissues and uncontrolled differentiation as well as obstacles such as poor transport efficiency to target tissues remain ongoing challenges for MSC therapies (Fischer et al., 2009; Liu Z. et al., 2018).

### Extracellular Vesicles Represent an Alternative to Stem Cells in Bone Regeneration

Extracellular vesicles, which are a type of nanometer-scale (30–2000 nm) heterogeneous particles, are vesicular particles released into the extracellular space by various cell types and act as protective carriers for DNA fragments, messenger RNAs (mRNAs), proteins, and lipids (Malda et al., 2016; Geeurickx et al., 2019). Characterized by excellent biocompatibility, long-term stability, and low immunogenicity, EVs have attracted extensive interest, especially in the field of bone tissue remodeling (Liu M. et al., 2018).

**FIGURE 2:**
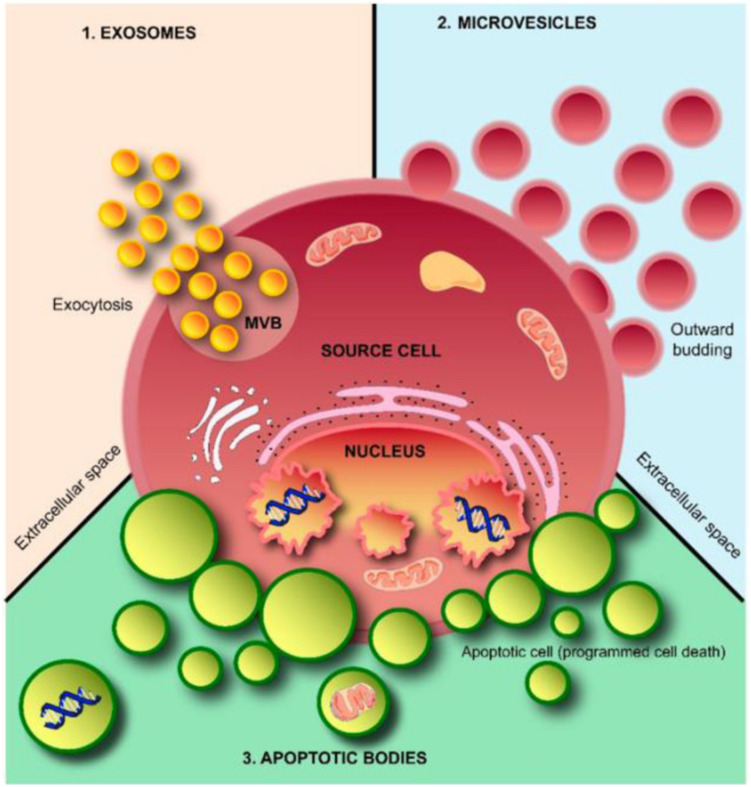
Schematic representation of extracellular vesicles (EVs). EVs are released from cells through outward budding of the plasma membrane (microvesicle pathway), exocytosis (exosome pathway), or apoptosis by dying cells (apoptotic body pathway). Adapted from Kalra et al. (2016).

According to their diameters, morphology, and biological characteristics, EVs can be further classified into three broad subpopulations, exosomes, microvesicles (MVs), and apoptotic bodies (ABs) (Table 1; Andaloussi et al., 2013; Kalra et al., 2016; Shao et al., 2018). Exosomes (40–120 nm) are cup-shaped vesicles derived from the endolysosomal pathway and are formed from multivesicular bodies when multivesicular bodies fuse with the cytoplasmic membrane. MVs (50–1000 nm) are formed through budding of the membrane and shuttle local cytosolic biomolecules. Larger ABs (500–2000 nm) are released during the cell apoptotic process and contain cell debris, organelles, and nuclear particulates derived from karyorrhexis (Figure 2).

**TABLE 1:** Characteristics of three major subtypes of EVs.

**EV**	**Size**	**Shape**	**Marker**	**Origin**
Exosome	40–120 nm	Round	Tetraspanins, Alix, TSG101, PDCD6IP, flotillin, MFGE8	Endolysosomal pathway
Microvesicle	50–1000 nm	Irregular	Integrins, MMPs, selectins, CD40	Plasma membrane
Apoptotic body	500–2000 nm	Heterogeneous	Phosphatidylserine, genomic DNA	Plasma membrane, endoplasmic reticulum

EVs can be derived from various types of MSCs. It has been reported that EVs show features similar to those of their parent MSCs, such as immunomodulatory and regenerative potential, and the low number of limitations for cell-free therapies make EVs an impressive option for tissue regeneration (Crivelli et al., 2017; Lou et al., 2017; Erten and Arslan, 2018). EVs could pass through capillaries more easily than MSCs, whose sizes in the circulation are too large for passage, and a proportion of EVs can even integrate into the perivascular niche (Toma et al., 2009). The dose of infused MSCs could diminish quickly post-transportation, whereas EVs remain at a relatively high concentration (Phinney and Pittenger, 2017). Moreover, EVs show significant osteogenic inductive potential, and EVs derived from umbilical cord mesenchymal stem cells were reported to be able to enhance the osteogenic capacity of pluripotent stem cell-derived MSCs, bone marrow mesenchymal stem cells (BMMSCs), human adipose mesenchymal stem cells, and other stem cells (Jia et al., 2019; Presen et al., 2019; Xu et al., 2019). These results have led to an increasing number of studies on EVs in bone regeneration medicine.

## Application of EVs in BTE

### The Cargoes of EVs Facilitate Bone Regeneration

EVs contain essential biomolecules, including lipids, membrane proteins, and other cell-specific proteins as well as several types of nucleic acids (Riazifar et al., 2017). The cargoes of EVs related to bone formation can be divided into canonical species and special species, as reviewed previously (Andaloussi et al., 2013). Canonical species are involved in the biogenesis or transport of vesicles, such as cytoskeletal proteins, specific stress proteins, and enzymes. The special cargoes in bone-related EVs, as a reflection of parent cell function, consist of specific osteogenetic proteins and non-collagenous matrix proteins, such as bone morphogenetic protein (BMP), alkaline phosphatase (ALP), eukaryotic initiation factor 2 (eIF2), osteopontin (OPN), osteocalcin (OCN), and osteonectin (ON) (Xiao et al., 2007). EVs also contain cargoes related to osteoclast differentiation, such as receptor activator of nuclear factor kappa-B (RANK) and receptor activator of nuclear factor kappa-B ligand (RANKL) (Deng et al., 2015; Huynh et al., 2016; Li D. et al., 2016). MicroRNAs (miRNAs) are another essential component of EVs, and miRNAs contributing to osteogenic capability include miR−196a, miR−27a, miR−206, miR-24, miR-143-3p, miR-10b-5p, miR-199b, and miR-218 (Liu M. et al., 2018). Moreover, mRNAs, as another essential component transported in EVs, are also involved in transcription (BDP1, TAF7L, and SOX11) and kinase activity (LPAR1 and ZEB2) (Qin et al., 2016, 2017; Morhayim et al., 2017; Lv et al., 2019).

### EV-Mediated Bone Regeneration

EVs are involved in the bone repair and regeneration process (Stroncek and Reichert, 2008), including the regulation of immune environments, enhancement of angiogenesis, differentiation of osteoblasts and osteoclasts, and promotion of bone mineralization.

EVs function as immunomodulatory messengers to mediate immune stimulation or suppression (Silva et al., 2017). EVs derived from MSCs (MSC-EVs) deliver several immune modulators, including programmed death ligand-1 (PDL-1), galectin-1, and TGF-β, which were reported to exert tolerogenic effects similar to those of MSCs (Mokarizadeh et al., 2012). Furthermore, various MSC-EVs could differentially regulate the expression of CD45RA on CD4^+^ or CD8^+^ T cells, resulting in a shift in the frequency of T cell subsets (Kordelas et al., 2019). M2 macrophages, which have an anti-inflammatory phenotype, are responsible for immune regulation and tissue remodeling. Studies have been performed to certify that MSC-EVs can polarize monocytes toward the M2 phenotype (Zhang et al., 2014; Chu et al., 2017).

EVs could also improve bone regeneration by enhancing angiogenesis. A recent study showed that UCMSC-derived exosomes accelerated angiogenesis and bone repair by promoting endothelial cell proliferation, migration and tube formation (Zhang et al., 2019). Exosomes secreted from iPSC-MSCs were proven to play a role in critical-sized bone defect repair in osteoporotic rats, and osteonecrosis prevention in the femoral head *via* the enhancement of angiogenesis and osteogenesis was also observed (Zhang et al., 2016; Liu X. et al., 2017a).

Studies revealed that EVs were able to promote the differentiation of osteoclasts, osteoblasts and BMMSCs, helping to maintain the balance of bone metabolism (Deng et al., 2015; Liu J. et al., 2017; Xie Y. et al., 2017). As was observed previously, bone remodeling was found to be performed continuously by osteoclasts *via* mineralized bone resorption and by osteoblasts *via* bone matrix synthesis. Both osteoblasts and osteoclasts can regulate bone homeostasis through paracrine signaling mediated by EVs (Ge et al., 2015; Li Q. et al., 2018). It was also proven that miRNAs of osteoclast-derived exosomes exerted inhibitory effects on bone formation (Li D. et al., 2016). Moreover, EV-associated senescence was shown to disrupt the balance of bone metabolism (Davis et al., 2017; Park et al., 2017).

Additionally, bone mineralization is also an essential process in bone generation, during which a specialized type of EVs known as the matrix vesicles that are present in the growth plate of developing bone has been shown to play an important role and has attracted increasing attention. Matrix vesicles are considered the initial site of the mineral formation of the newly formed bone matrix *via* hydroxyapatite deposition (Golub, 2009). Calcium and phosphate are transported to initiate formation as well as accumulation of hydroxyapatite crystals in matrix vesicles, and then these crystals are released into the extravesicular fluid to guide calcification following collagen calcification (Chu et al., 2019). In addition, osteoblast-derived EVs were reported to mediate mineralization dynamically along with alterations in vesicle morphology and content, leading to developmental changes during matrix organization (Davies et al., 2019).

**FIGURE 3:**
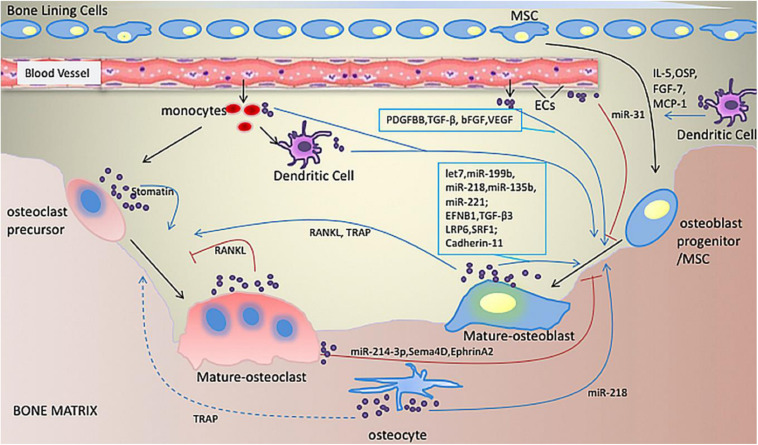
Illustration of bone-related EVs. EVs play roles in communication between cells related to bone formation. Various miRNAs and proteins in EVs derived from osteoblasts, osteoclasts, osteocytes, monocytes, macrophages and dendritic cells are involved in enhancing or inhibiting osteogenic activity. Adapted with permission from Jin et al. (2019).

In brief, although the underlying mechanisms of the osteogenic effects of EVs remain unclear, EVs have been shown to play a role in cellular signaling and the molecular transport of bone by regulating the immune microenvironment, promoting angiogenesis, balancing bone metabolism, and participating in mineralization. Furthermore, their protective effects in hypoxic and ischemic conditions (Figure 3; Hugel et al., 2005; Liu X. et al., 2017a; Jin et al., 2019), and endogenous MSC recruitment in bone regeneration cannot be ignored (Furuta et al., 2016).

### The Challenges of EVs Therapy

The abilities of EVs to carry and protect various encapsulated molecules make them a promising option for therapeutic applications. Currently, EVs therapy attempts have included the treatment of cancer, neurodegenerative disorders, and cardiovascular disease and tissue repair and regeneration. However, in EVs therapy, species specificity, immunogenicity and unpredictable effects have contributed to difficulties not only in terms of reproducibility, stability and purity but also safety and toxicity testing.

For either local or systemic delivery, one major obstacle for the usage of EVs is that naked EVs fail to attain a suitable concentration for therapeutic needs. Off-target effects, short-term retention in the tissue site, and accumulation in non-targeted organs are also inevitable (Imai et al., 2015; Gualerzi et al., 2017; Mollaei et al., 2017). Thus, increased doses of EVs have been considered to resist the weakening effects. However, syngeneic exosome injection caused the rapid asphyxiation of mice due to the overaccumulation of EVs in the lungs despite all the other positive effects (Smyth et al., 2015). Furthermore, studies with the aim of facilitating EVs tropism provide another possible solution, but it also remains a challenge to determine the surface proteins responsible for the binding of ligands to different targets. In order to overcome these challenges, a range of sufficient scaffolds for loading, protecting, and controlling the release of EVs are in needed.

## Applications of Biomaterial-EV Delivery Systems in BTE

**FIGURE 4:**
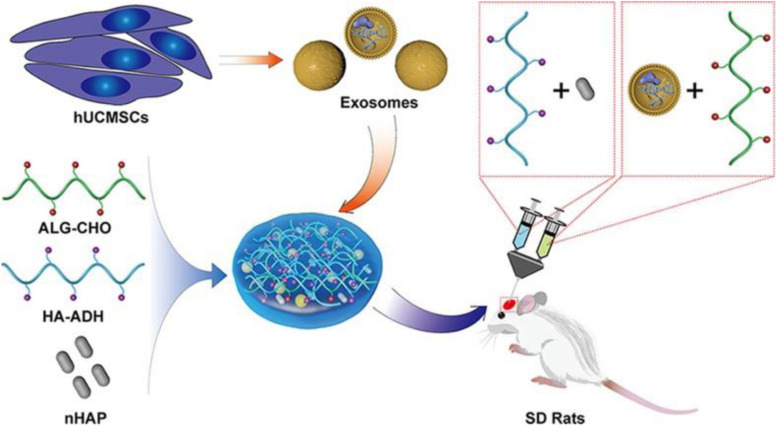
Schematic illustration of the EV/scaffold study design for bone defect repair. An injectable HAP-embedded *in situ* cross-linked HA-ALG hydrogel system was synthesized to facilitate controlled delivery and mechanical support of EVs. The exosomes were isolated from hUCMSCs and then incorporated into the HA-ALG hydrogel scaffold system in a rat model of calvarial bone defects in vivo to reveal bone regeneration and angiogenesis effects. Reproduced with permission from Yang et al. (2020). hUCMSCs, human umbilical cord mesenchymal stem cells; HAP, hydroxyapatite; HA-ALG, hyaluronic acid-alginate; HA-ADH, hyaluronic acid- adipic dihydrazide; ALG-CHO, aldehyde-modified alginate; SD rats, Sprague Dawley rats.

Since naked EVs are vulnerable when transplanted *in vivo* and difficult to target to bone defect sites, the approach of loading EVs with biomaterial systems possesses tremendous advantages, as shown in the schematic illustration in Figure 4. In fact, EVs delivered by biomaterials are the promising tools for bone regeneration. They could be adhered by gels, bound actively to molecular linkers or attached to the surfaces of biomaterials to permit the controlled release of EVs (Silva et al., 2017).

**FIGURE 5:**
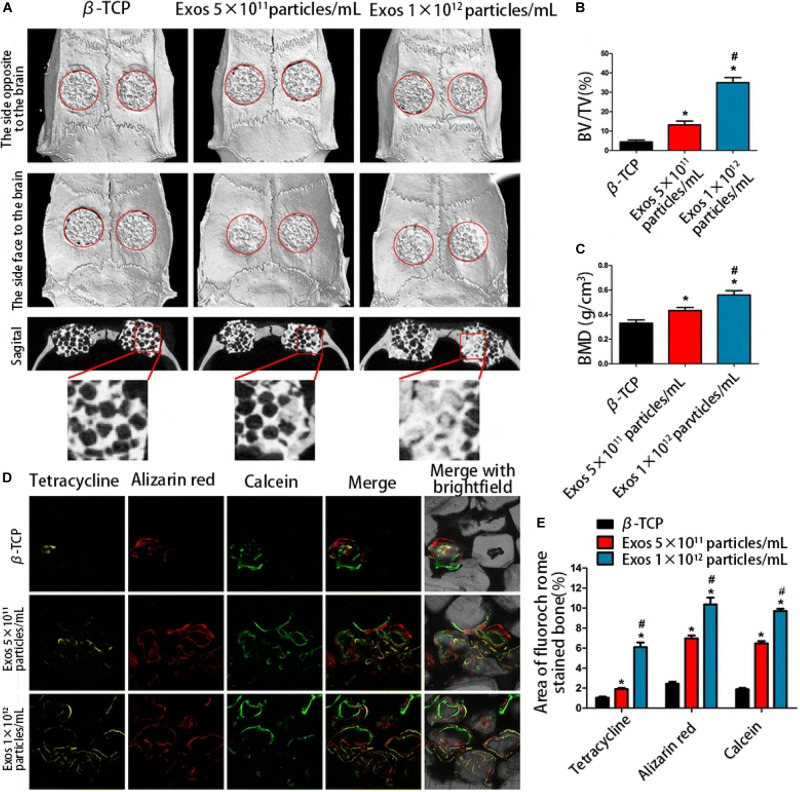
The enhanced osteogenic effect of exosome/β-TCP scaffolds. Exosomes derived from human-induced pluripotent stem cell-derived mesenchymal stem cells were loaded with β-TCP, and the osteogenesis effect in a rat model of critical-sized calvarial bone defects was evaluated at 8 weeks post-operation. **(A)** The different effects on repair of β-TCP alone or different concentrations of exosomes on three-dimensional reconstruction and sagittal images. **(B,C)** BV/TV and BMD differed between groups. **(D)** The formation and mineralization effect of new bone at 8 weeks post-operation was identified *via* fluorochrome-labeling histomorphometrical analysis. **(E)** Proportion of the fluorochrome area for all groups. The exosome/β-TCP system was shown to facilitate bone repair more remarkably than pure β-TCP scaffolds. Reproduced from Zhang et al. (2016). β-TCP, tricalcium phosphate; Exo, exosome; BV/TV, the ratio of bone volume to tissue volume; BMD, the bone mineral density.

Several studies have indicated that enhanced bone regeneration was achieved by scaffold-mediated EVs therapies in bone defect models by using bioceramic, polymer, and composite scaffolds (Table 2; Qin et al., 2016; Zhang et al., 2016, 2019; Li W. et al., 2018; Chen et al., 2019; Wu et al., 2019; Yang et al., 2020). The exosome/β-TCP combination systems were embedded into the defect in a rat critical-sized calvarial bone defect model, leading to better osteogenesis ability than the use of β-TCP scaffolds alone (Figure 5; Zhang et al., 2016), and Wu et al. (2019) also achieved similar results with an exosome/β-TCP system in alveolar bone regeneration. In another study, Li W. et al. (2018) achieved the accelerated restoration of calvarial bone defects in a mouse model by integrating exosomes derived from human adipose-derived stem cells (hASCs) with polydopamine (PDA)-coated poly (lactic-coglycolic acid)/(PLGA) scaffolds, which resulted in timed release and enhanced bioactivities. This *in vitro* cell-free system showed that stimulated osteoinductive effects were favorable in improving the proliferation, migration and homing of MSCs in new bone. Qin et al. (2016) constructed an EVs delivery system with a commercially available hydrogel (HyStem-HP) to accelerate bone regeneration in a rat critical-sized calvarial bone defect model. BMMSC-EVs were demonstrated to enter osteoblasts and deliver osteogenic miRNAs by endocytosis and thus to modulate osteogenesis-related gene marker expression and hence differentiation *in vitro*, and hydrogels loaded with EVs enhanced bone formation *in vivo*, where miR-196 might be involved in bone regeneration. It is interesting to note that the role of osteogenesis-relevant molecules has been determined to improve the efficacy of EVs therapies. Chen et al. (2019) used commercial hydrogels (Glycosan Biosystems) embedded with hAD-MSC-derived EVs that overexpressed miR-375 to evaluate the effect on bone regeneration. The engineered hydrogel displayed a slow and sustained exosome release process, and *in vivo* studies showed that exosomes (miR-375) loaded with hydrogel promoted bone regeneration in calvarial defects in a rat model. In the study carried out by Zhang et al. (2019), effects on angiogenesis and bone repair were observed in a rat model of femoral fracture by applying hydrogel (HyStem-HP) incorporating uMSC-derived exosomes. The positive effects of uMSC-derived exosomes on enhancing bone fracture repair may be attributed to the upregulation of HIF-1α and the control of VEGF gene expression during angiogenesis. However, the effect of exosomes on enhancing osteoblast differentiation was not observed in a subsequent *in vitro* study. Qayoom et al. (2019) also proposed the role of EVs in a femur neck canal defect model in osteoporotic rats as a biological carrier facilitating bone formation and controlling the dosage of BMP. They used nano cements to deliver BMPs, exosomes, and bisphosphonates, which improved biomechanical strength in the defect. Recently, Yang et al. (2020) isolated hUCMSC-derived exosomes and fabricated an injectable HAP-incorporating *in situ* cross-linked hyaluronic acid-alginate (HA-ALG) hydrogel scaffold to realize the controlled delivery of exosomes as well as the physical support of defects. The release rate of exosomes from the system was approximately 71.2%, and the integration of exosomes and the hydrogel also resulted in a bone reparative effect in a rat calvarial bone defect model.

**TABLE 2:** Summary of the scaffold-mediated EVs delivery system for bone regeneration.

**Source/type of EVs**	**Carrier scaffolds**	**Experiment procedure**	**Findings**	**References**
EVs derived from human BMMSCs	Hydrogel	• *In vitro* • *In vivo:* rat calvarial bone defect	• BMSC-derived EVs regulate differentiation of osteoblast and expression of osteogenic genes *in vitro.* • Bone formation is enhanced *in vivo.* • Exogenous EVs enter the Golgi apparatus.	Qin et al., 2016
Exosomes derived from human iPSC-MSCs	β-TCP scaffold	• *In vitro* • *In vivo:* rat calvarial bone defect	• The exosomes internalized by hBMSCs could profoundly enhance the proliferation, migration, and osteogenic differentiation of hBMSCs. • Osteogenesis of the exosomes + β-TCP combination scaffold was promoted *in vivo* as compared to β-TCP alone.	Zhang et al., 2016
MVs derived from rat BMMSCs	Alginate-PCL scaffold	• *In vitro* • *In vivo:* a subcutaneous bone formation model in nude mice	• MSC-MVs enhance capillary network formation of HUVECs *in vitro.* • MVs+ alginate-PCL scaffold increased vascularization and tissue-engineered bone regeneration *in vivo.*	Xie H. et al., 2017
Exosomes derived from hASCs	PLGA/pDA scaffold	• *In vitro* • *In vivo:* Hind limb ischemia in murine model	• Exosomes could promote osteogenesis, proliferation, and migration effects of hBMSCs. • Exosomes were released from PLGA/pDA scaffold under control. • Osteoinductive effects and migration and homing of MSCs were enhanced *in vivo.*	Li W. et al., 2018
Exosomes derived from human BMMSCs	Calcium sulfate/nanohydroxyapatite-based NC	• *In vivo*: • osteoporosis in femur neck canal defect model	• NC work as a carrier to deliver drugs and other bioactive molecules. • A trend of promoted mechanical properties in the NC + BMP + ZA group was shown. • Exosomes enhance the bone formation in the absence of BMP.	Qayoom et al., 2019
Exosomes derived from hAD-MSCs	PLA-based CaSi-DCPD-doped scaffold	• *In vitro*	• The PLA-based scaffolds could adhere, keep and release exosomes. • The osteogenic properties of hAD-MSCs were promoted by the EV-enriched scaffold. • Mineral-doped scaffolds stimulated osteogenesis of hAD-MSCs, and showed a potential in regenerative bone healing.	Gandolfi et al., 2020
Exosomes derived from human MSCs	3D collagen hydrogels	• *In vitro* • *In vivo*: athymic nude mice	• Exosomes derived from osteogenic hMSCs trigger lineage specific differentiation of naive hMSCs both *in vitro* and *in vivo*. • Exosomes can bind to ECM proteins like type I collagen and fibronectin.	Narayanan et al., 2016
Exosomes derived from hPDLSCs	Collagen membrane (Evolution), PEI-modified	• *In vitro* • *In vivo*: rat calvarial bone defect	PEI-EVs promoted osseointegration activity by enhancing mineralization and vascular network. • The system could induce bone regeneration.	Diomede et al., 2018a
Exosomes derived from human gingival MSCs	3D-PLA scaffold, PEI-modified	• *In vitro* • *In vivo*: rat calvarial bone defect	PEI-EVs play a role in activating local bone induction. • The system facilitates bone repair by enhancing mineralization and vascularization.	Diomede et al., 2018b

*β-TCP, tricalcium phosphate; PCL, alginate-polycaprolactone; MVs, microvesicles; HUVECs, human umbilical vein endothelial cells; hASCs, human adipose-derived stem cells; PLGA/pDA, polydopamine-coating poly (lactic-co-glycolic acid); NC, nanocement; ZA, zoledronate; BMP, bone morphogenetic protein; hAD-MSCs, human adipose mesenchymal stem cells; PLA, polylactic acid; CaSi, calcium silicate; DCPD, dicalcium phosphate dihydrate; hPDLSCs, human periodontal-ligament stem cells; PEI, polyethylenimine.*

Additionally, a demineralized bone matrix (DBM) with a certain 3-dimensional (3D) structure with specific osteogenic induction ability and tissue biocompatibility was fabricated. Xie H. et al. (2017) functionalized DBM scaffolds with BMMSC-derived MVs and demonstrated that increased angiogenic and osteogenic effects were observed in a model of ectopic subcutaneous bone formation in nude mice, whereas decalcification could also cause a deficiency in mechanical strength and stress (Marcos-Campos et al., 2012; Li et al., 2013). Therefore, to achieve improved osteogenic properties, modifications of EV/biomaterial osteogenic systems need to be further explored.

## Methods for the Modification of Biomaterials to Facilitate EVs Applications in BTE

Current modifications to improve the osteogenic effect of EV/scaffold treatment can be broadly divided into two categories. One category focuses on the process of loading EVs into scaffolds to achieve efficient transportation to defect sites and controlled release of EVs. The other category involves the interaction between EV/scaffold and the microenvironment, which would affect signal regulation and the proliferation and differentiation of stem cells to repair bone injuries. It has been widely accepted that the biological behaviors of cells as well as those of tissues are associated with the design and properties of biomaterials (Li et al., 2017). More importantly, some functions of biomaterials are influenced by biological responses, and the characteristics of biomaterials themselves would offer biological cues to modulate cell behaviors and ultimately increase tissue regeneration. Herein, we summarize modification methods described in recent studies to facilitate EVs delivery efficiency in BTE in terms of three main aspects.

### Pore Parameters

In EV-mediated cell-free therapy, appropriate pore parameters are necessary to sustain the controlled release ability of scaffolds. The pore sizes are relevant to cell migration and proliferation, and the increase in porosity leads to enhanced permeability as well as the loss of mechanical properties. Although there is still no consensus on the specific optimal values of pore size or porosity, a higher porosity (more than 90%) with a range of pore sizes (10–200 μm) is recommended to maintain proper mechanical properties (Boyan et al., 2002; Perez and Mestres, 2016). Liu X. et al. (2017b) reported the use of EVs encapsulated in a photoinduced imine crosslinking hydrogel glue and tested the exosome retention ability of the exosome-complexed hydrogel tissue patch (EHG). Based on classical rubber theory, the pore size of the hydrogel was theoretically 25 nm, which was smaller than that of exosomes, allowing the majority of the exosomes to be retained inside the scaffold. This indicated that EHG could effectively retain exosomes inside the hydrogel, and over 1 × 10^10^ mL^–1^ of exosomes per day were released, resulting in the acceleration of cartilage defect repair (Liu X. et al., 2017b). Another study by Gandolfi et al. (2020) produced mineral-doped PLA-based scaffolds enriched with EVs to evaluate the osteogenic effects on hAD-MSCs. They showed a dynamic change after depositing exosomes on the surface of the scaffold; as the PLA matrix partially degraded, the formation of a calcium phosphate layer partially filled the pores and led to a decrease in porosity, which influenced the biological behavior of the hAD-MSCs.

### Electrostatic Interaction

Another attractive aspect of modifying EVs delivery systems is based on electrostatic interactions. The charge and potential of EVs membranes could affect their interactions with biomaterials (Gerlach and Griffin, 2016). Studies have shown that the average potential of exosomes is approximately 40 mV, and the negatively charged phospholipid membranes of EVs are responsible for their negatively charged status (Sokolova et al., 2011; Charoenviriyakul et al., 2017). In addition, the charged residues carried by the EVs glycocalyx also influence the interaction of EVs with biomaterials through attraction or repulsion (Gomes et al., 2015; Gerlach and Griffin, 2016). In applications in bone regeneration, polyethylenimine (PEI), as a positively charged biocompatible polymer with low toxicity and high biological activity, has been exploited in the engineering of negatively charged EVs (Werth et al., 2006). Diomede et al. (2018b) employed PEI to enhance the adhesion of EVs onto a 3D PLA biomaterial to regenerate bone defects induced in rat calvaria. The results suggested that the number of EVs inside the cells of the PEI-EV group was higher than that of the non-engineered-EV group, which could be ascribed to the use of cationic PEI to favor internalization *via* proteoglycan binding. The team also designed a biocompatible osteogenesis system composed of collagen membranes (Evolution [Evo]) and hPDLSCs enriched with PEI-EVs, and the similar results indicated that PEI-EVs were involved in activating osteogenesis (Diomede et al., 2018a).

### Bioactive Adhesion

Bioactive adhesion between EVs and scaffolds is an important factor in achieving ideal bone regeneration. MSC-EVs were verified to express several adhesion molecules found in MSCs, including CD44, CD29 (β_1_-integrin), CD73, and α_4_- and α_5_-integrins (Bruno et al., 2009). Narayanan et al. (2016) found that MSC-EVs could combine with extracellular matrix proteins such as type I collagen and fibronectin, which are the two main components of the ECM. Therefore, fibronectin-coated DBM scaffolds were generated before loading EVs to enhance the adherence of EVs to the biomaterials. EVs were shown to remain adherent for a longer time and to be released evenly after several washes with phosphate-buffered saline, and the proangiogenic and pro-osteogenesis activities of the EV-modified scaffolds were also verified (Xie H. et al., 2017). Likewise, PDAs employed in tissue engineering have the specific ability to produce high adherence in anchoring substances onto substrates (Ho and Ding, 2014). Li W. et al. (2018) proved that the use of a cell-free osteogenic system with PLGA/pDA scaffolds to immobilize exosomes was effective in promoting the migration and homing of hASCs and osteogenic induction *in vivo*.

## Limitations and Future Perspectives

EVs exert beneficial effects on bone regeneration, facilitating osteogenesis and enhancing mineralization as well as vascularization. As an emerging tool for cell-free therapies, EVs have attracted great attention and have been tested in numerous *in vivo* and *in vitro* studies. However, most clinical trials of EVs have been focused on the treatment of cancer and nervous system diseases (Chung et al., 2020), while few trials of EVs in bone regeneration have been performed. To date, only one clinical study (ClinicalTrials.gov, Identifier: NCT04281901) of bone disease involving EVs has been performed, in which extracellular vesicle-rich plasma (PVRP) was compared with platelet-rich plasma (PRP) for the treatment of chronically inflamed postsurgical temporal bone cavities. One of the reasons that EV-involved BTE products are still far from ideal for use in clinical applications and industrial production is that there is currently no consensus on the best method of enrichment and purification, and the effective concentration of EVs among current studies in the field of regeneration have not been clarified. Furthermore, as various biomaterials have been created, the modification of scaffold design and production techniques have tremendously boosted the efficiency of bone regeneration. Therefore, EVs loaded in bioscaffolds have the considerable potential to bypass a critical bottleneck of traditional therapies for bone defects. Some potential evidence from fields besides bone regeneration indicates that it is possible to control EVs to be delivered by scaffolds *via* pH-response release systems based on the Schiff base reaction (Cardoso et al., 2015; Wang et al., 2019), the use of an electrospun nanofibrous reservoir layer with hydrophobic properties and ionic interactions based on the electrospinning technique (Mašek et al., 2017), and aquaporin-mediated EVs deformability in water permeation (Fuhrmann, 2020; Lenzini et al., 2020). Since the effects of scaffolds loaded with EVs are influenced not only by the active components and adhesion but also the degradation rate, stress distribution and mechanical properties of scaffolds, the standardization of manufacturing processes as well as the application of regulations remain to be clarified. In conclusion, a better understanding of the promise of EVs therapy will be obtained when a body of evidence from preclinical and clinical studies of biomaterial-mediated EVs therapies is carefully taken into account.

## Author Contributions

D-WL and T-TY organized the text and content and edited and modified the manuscript. H-CY wrote the main manuscript text and prepared the figures. JL, Y-QQ, L-CW, TZ, QL, and Y-HZ reviewed the manuscript. All authors have approved the final version of the manuscript.

## Conflict of Interest

The authors declare that the research was conducted in the absence of any commercial or financial relationships that could be construed as a potential conflict of interest.
